# Considerations in Designing Digital Peer Support for Mental Health: Interview Study Among Users of a Digital Support System (Buddy Project)

**DOI:** 10.2196/21819

**Published:** 2021-01-04

**Authors:** Nazanin Andalibi, Madison K Flood

**Affiliations:** 1 University of Michigan School of Information Ann Arbor, MI United States; 2 Department of History of Art University of Michigan Ann Arbor, MI United States

**Keywords:** mental health, peer support, technology, design, digital peer support, mHealth, digital health, internet

## Abstract

**Background:**

Peer support is an approach to cope with mental illness, and technology provides a way to facilitate peer support. However, there are barriers to seeking support in offline and technology-mediated contexts.

**Objective:**

This study aims to uncover potential ways to design digital mental health peer support systems and to outline a set of principles for future designers to consider as they embark on designing these systems. By learning how existing systems are used by people in daily life and by centering their experiences, we can better understand how to design mental health peer support technologies that foreground people’s needs. One existing digital peer support system is Buddy Project, the case study in this paper.

**Methods:**

This paper reports on an interview study with Buddy Project users (N=13). Data were analyzed using the constant comparative approach.

**Results:**

Individuals matched through Buddy Project developed supportive friendships with one another, leading them to become each other’s peer supporters in their respective journeys. It was not only the mental health peer support that was important to participants but also being able to connect over other parts of their lives and identities. The design of Buddy Project provided a sense of anonymity and separation from pre-existing ties, making it easier for participants to disclose struggles; moreover, the pairs appreciated being able to browse each other’s social media pages before connecting. Buddy Project has an explicit mission to prevent suicide and demonstrates this mission across its online platforms, which helps reduce the stigma around mental health within the peer support space. Pairs were matched based on shared interests and identities. This choice aided the pairs in developing meaningful, compatible, and supportive relationships with each other, where they felt seen and understood. However, the pairs were concerned that matching based on a shared mental health diagnosis may lead to sharing unhealthy coping mechanisms or comparing themselves and the severity of their experiences with their peers.

**Conclusions:**

The results of this study shed light on desirable features of a digital mental health peer support system: matching peers based on interests and identities that they self-identify with; having an explicit mental health–related mission coupled with social media and other web-based presences to signal that discussing mental health is safe within the peer support ecosystem; and not matching peers based on a broad mental health diagnosis. However, if the diagnosis is important, this matching should account for illness severity and educate peers on how to provide support while avoiding suggesting unhelpful coping mechanisms; allowing for some degree of anonymity and control over how peers present themselves to each other; and providing relevant information and tools to potential peers to help them decide if they would like to embark on a relationship with their matched peer before connecting with them.

## Introduction

Mental health is a topic of utmost importance to public health. Nearly 20% of US adults live with a mental illness, with the majority never accessing care [1]. Every day, an average of 129 Americans die by suicide [2]. In 2017, it was estimated that 18.9% of all US adults had a mental, behavioral, or emotional disorder [1]. More specifically, young adults aged from 18 to 25 years had the highest prevalence of mental illness (25.8%) among other age groups; however, the percentage of young adults who received mental health services was lower (38.4%) than adults of other age groups [1]. Although young adults tend to be one of the most vulnerable groups with respect to mental illnesses, their mental health care needs often remain unmet [3]. As a result of mental illness, suicidal ideations are estimated to affect 25% of young adults, with helplessness being the most reported motive [4].

The major barriers to accessing and seeking mental health care include stigma associated with mental illness, shortage of trained professionals, treatment costs, concerns about confidentiality, lack of knowledge of resources, and inaccessibility of services [5,6]. Furthermore, the United States is projected to have shortages in most types of mental health care providers by 2025 [7]. As such, new approaches that can complement or expand the capacity of mental health care are needed. One such approach is to facilitate seeking and finding social and peer support.

Social support and peer support are well-established approaches to cope with illnesses, including mental illness [8]; however, when struggling with mental illness, seeking and finding appropriate support is difficult [9]. One key mechanism through which social and peer support may be accessed is through technology and the internet [10]. Peer support, typically exchanged between individuals who share an experience, can facilitate accessing social support by providing information support regarding professional help [11] and emotional and esteem support by boosting one’s self-esteem [11], increasing hope [12], reducing the feeling of isolation [13], and allowing peers to feel that they belong to a community [14].

Previous work [10,11,14-16] has established the benefits of creating technology-mediated support systems, including the option to use pseudonyms, an opportunity for social interaction, increased accessibility to a wide range of coping mechanisms and support tools, an increased likelihood of seeking help, a better chance of finding a destigmatized conversation space, and being part of a positive and supportive community of similar individuals. In addition, individuals experiencing mental illness often face unique barriers to build friendships or other social connections [15,17]. An example is social anxiety, which may lead to lower friendship quality or the fear of being rejected or embarrassed [18]. Therefore, using the internet as a way to develop these social connections has proved to be helpful. More broadly, young adults are reluctant to seek professional face-to-face help for their mental health conditions [19]. Therefore, examining other mechanisms that might be helpful to their coping and engagement with peer support is important.

Reports show that approximately 90% of American adults use the internet [20]. In fact, a growing portion of young adults use the internet to access mental health resources [21]. The moment an individual takes the first step to find support and connect to others is a critical point in their illness journey [22], whether that is seeking peer support or other types of care. However, with some exceptions [15], design guidelines for mental health technologies have mostly focused on systems that bring individuals with mental illness in contact with their providers rather than peers [23]. In fact, technology design for peers to support each other has lagged behind other technological innovations for mental health [24]. Although we know that peer support is helpful in coping with and managing mental illness [16] and that technology has the potential to facilitate this coping process [13], there remains a need to interrogate existing peer support systems for their success or lack thereof and learn from them to contribute to our knowledge about how technology-mediated mental health peer support may be designed. By learning how existing systems are used by people in their natural settings and by centering on their experiences, we can better understand how to design peer support technologies that center people’s needs. We address this gap by taking Buddy Project [25], an online peer support system for mental health and a nonprofit organization, as a case study. Buddy Project is a peer support system interested in fostering friendship, peer support, and connection, which is one aspect of mental health care. Buddy Project is not a replacement for formal treatment or a place to monitor mental health.

### Buddy Project

**Figure 1:**
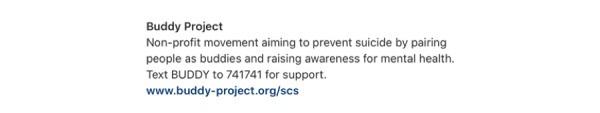
Buddy Project mission statements on Instagram biography.

**Figure 2:**
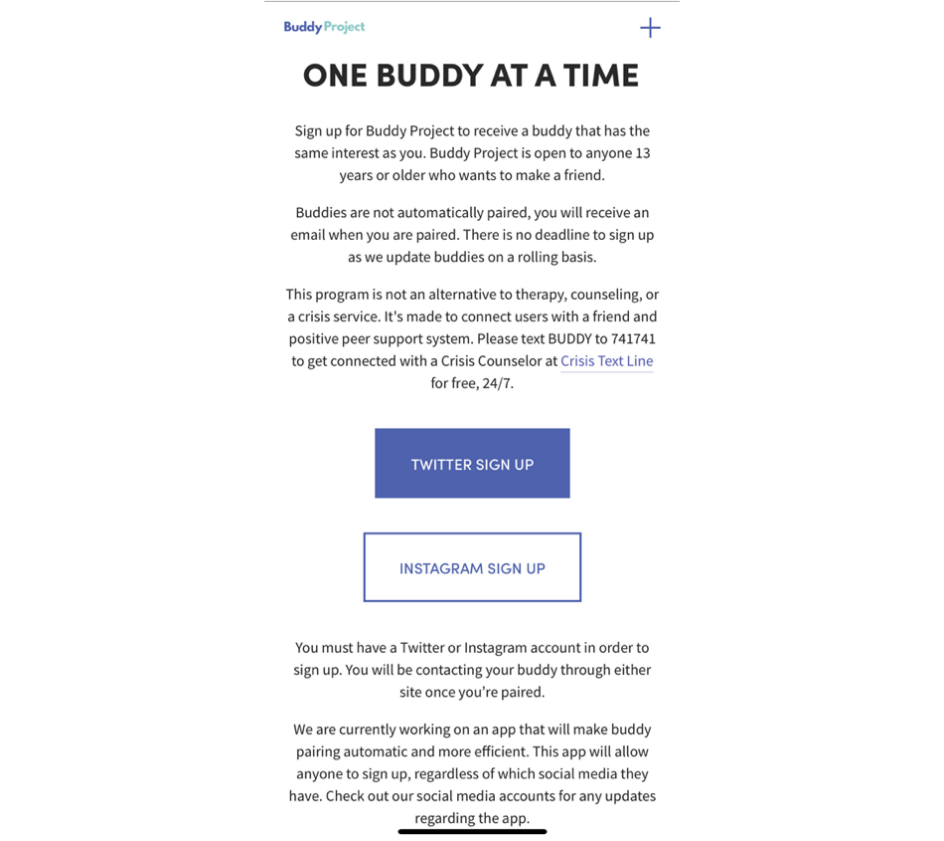
The sign-up page on the Buddy Project website.

For this study, we turned to Buddy Project, an online peer-to-peer support system that aims at fostering relationships that provide support to those struggling with mental illness. Buddy Project was founded in 2015 by Gabby Frost, a young person who wanted to prevent suicide and self-harm while advocating for mental health. Buddy Project’s mission to prevent suicide is now displayed on the website’s home page and in its Twitter and Instagram biographies (Figure 1). So far, the nonprofit movement has paired more than 236,000 adolescents aged between 12 and 25 years. Participants are matched based on age difference and shared interests and connected using their social media accounts (Twitter or Instagram). The sign-up process is shown in Figure 2, where users choose the social media account they want to use. Peers are then redirected to a Google form. On the first page, they provide their email address, first name, and Twitter or Instagram username, depending on the sign-up process they selected. On the second page, peers rank their first to fifth *interests*. A complete list of the interest options is included in Multimedia Appendix 1. We developed higher-level themes for these interests to gain a better sense of what they include. Interests categories include arts and entertainment (eg, musicians, television shows, books), identity (eg, religion, political ideologies, gender, and sexuality), and time zones. The third page prompts peers to provide their age. The last page informs peers what to expect once they submit the Google form. It states that they will receive an email once paired with their buddy, that their selection is not automatic, and that peers are welcome to sign up for multiple buddies, along with the links to Buddy Project’s website, Instagram, Twitter, and Facebook accounts.

Buddy Project’s founder manually pairs all of the buddies using a Microsoft Excel spreadsheet. When pairing buddies, they take into consideration the interests they selected when signing up and their age. If someone wants to refuse a buddy, they can simply do so by not connecting with them once paired, and they can sign up to be paired with someone else. There is currently no formal training offered to buddies because buddies sign up to make friends who understand them (and thus can provide them with peer support), not provide or receive any type of professional help or crisis counseling.

We conducted in-depth semistructured interviews with 13 Buddy Project users.

### Literature Review

#### Social and Peer Support for Mental Health

Social support is crucial for maintaining well-being [8]. Broadly defined, social support is characterized by meaningful interactions that provide some sort of support [26-28]. Social support can take many forms, such as emotional (communicating care and compassion), esteem (communicating confidence in one’s ability), informational (providing information, advice, and tips), network (communicating that one is not alone and that there are many others who understand them), and tangible or instrumental support (providing tangible help and services) [29]. Receiving adequate and appropriate social support improves mental health and well-being [30]. For example, emotional and esteem support are successful in reducing the impact of a negative, unhealthy view of oneself by providing individuals with a higher sense of self-worth [31].

Gaining mental health support poses challenges because it can be time consuming, draining, and costly, and those affected by mental illness may have additional personal or social barriers to build friendships or connections [22]. For example, some may feel uncomfortable or unequipped to approach people or communicate in person to receive support or may have cognitive or social impairments, hindering their ability to seek and receive social support [22]. A main challenge in coping with mental illness is the stigma surrounding it, which is the idea that if one seeks or receives help, they are weak, incapable of taking care of themselves, or are inferior to people who can cope with mental illness on their own [11]. Stigma creates a barrier that prevents people from accessing or acquiring information about mental health resources [13]. Increasing communication about mental health–related topics, coping mechanisms, and common emotions reduces the perceived embarrassment associated with these conversations [32]. Although seeking help online can be beneficial in part because it can limit the consequences of stigma, there is still space to remove barriers to seeking help.

The dominant method of promoting mental health is providing clinical mental health services via professionals such as psychologists, psychotherapists, or social workers. However, most people with mental health disorders receive no treatment [33]. A complementary approach to seeking formal treatments is engaging in peer support. Peer support refers to the support that people with lived experience of an illness or condition (eg, mental illness) can provide to one another [34]. The key principles of peer support include respect, shared responsibility, and an agreement of what is helpful [35]. Peer support’s importance as a key recovery service for people with mental illnesses has been established globally [36].

#### Digital Peer Support and Mental Health

Technology is increasingly applied to deliver peer support to individuals with mental health conditions [37]. Digital peer support is defined as peer support mediated through technology [38]. Technologies such as support forums and groups, mental health–focused mobile apps, and more broadly social media, have the potential to facilitate finding social and peer support and coping with mental illness. Along with increasing the amount of social support one receives [39], peer support can complement other resources (eg, professional therapy) one may use to cope with mental illness. The support one can obtain from a trained professional is qualitatively different from the type of support that one can receive from similar others with comparable lived experiences [40].

Research has focused on developing and evaluating mental health apps. Mental health apps provide features such as information, monitoring medications and symptoms, telepsychiatry, cognitive behavioral therapy, and support groups [41]. However, interest in using mental health apps does not mean actual high usage of these apps [42-47].

Extant research also shows how people turn to online communities to seek and exchange social support with many similar others [48,49] and express themselves [50]. Some online spaces also afford one-on-one peer-to-peer support, which allows peers to connect over personal experiences [51]. Regardless of whether conversations are one-to-many (ie, one post from one person to many recipients) as in the case of forums or one-on-one as in the case of private chats, because of this shared experience, individuals feel more connected, less alone, and less ashamed of what they are going through [13]. Moreover, finding support online can be crucial for some more than others; for example, individuals with intersecting marginalized identities often find safe online communities *necessary* to receive social support [48]. Some degree of anonymity present in some peer support technologies (eg, allowing the use of pseudonyms rather than enforcing the use of physical world identities) removes barriers to seek support when facing stigma, making social support a more attainable coping resource for some in comparison with nonanonymous settings (eg, in other online spaces) [22].

Online and offline, peer-to-peer support unites individuals with mental health conditions, providing them the opportunity to engage in sharing experiences, feelings, coping mechanisms, advice, and support to improve their mental health condition [15]. Although traditional face-to-face communication cannot be replaced with technology [49], connecting with peers via the internet allows for widespread accessibility and often an easier way for users to engage with one another [13].

In summary, digital peer support for mental health is an emerging and promising research space that has the potential to help improve mental health conditions, self-management skill development, social functioning, hope, and empowerment [38]. A systematic literature review on digital peer support interventions found that digital peer support interventions are feasible and acceptable, with high potential for clinical effectiveness [52]. Furthermore, peer support has the potential to improve and change not just how we approach mental health but also social change more broadly [53].

It is important to learn from existing services and platforms that employ technology for mental health peer support in settings outside of controlled research studies (in the real world) to examine users’ perceptions and inform the design of future digital peer support systems. We turn to Buddy Project (described earlier) as a case study to do exactly that.

## Methods

### Recruitment

The recruitment process began with a screening survey that the Buddy Project organization shared on their Twitter and Instagram accounts. To participate in the survey, participants needed to (1) be a social media user, (2) live in the United States, (3) be at least 18 years old, and (4) be a current or previous user of Buddy Project. Being a social media user was important for us to situate Buddy Project within other online support systems that participants may have used. If they met these qualifications, participants were asked to complete the second portion of the survey that asked questions about demographics, social media use, length of longest relationship with buddies, number of buddies they had matched with, month and year when they first used Buddy Project, and overall experience with their buddies. We did not screen participants on the basis of mental health status or diagnosis, as we were interested in learning experiences with Buddy Project, and Buddy Project does not match buddies based on the diagnosis. The survey was open from June 12 to 25, 2019, and received 123 responses. From the responses, 63 participants met the initial criteria, 38 were invited to participate in the study, and 13 were interviewed. We sent an interview invitation to all qualified participants who had consistent survey responses (eg, the time of their longest relationship with a buddy did not extend past the date of when they started using Buddy Project) and had used Buddy Project within the last 2 years. We also purposefully recruited participants from diverse demographics to the extent possible. The invitation included study details and an online consent form. A total of 13 individuals completed these forms and participated in the interview. Table 1 includes the details of participants. We continued recruiting participants through the interview process and stopped when no new themes emerged. We offered a US $15 gift card to interview participants. This study was approved by our institutional review board.

Apart from assisting with recruitment, Buddy Project had no other role in any part of this study. We plan to share this paper with the founder after peer review.

**Table 1:** Participant details.

Participant^a^	Age (years)	Gender	Race	Education	Living area	Social media	User status	When they joined BP^b^	Total buddy count	Longest relationship	Overall experience
P1	19	Woman	Latina	Some college	Urban	TW^c^, SC^d^, TB^e^	Previous	2015	1	4 years	Both positive and negative
P2	19	Woman	Black	Some college	Urban	FB^f^, TW, IG^g^, SC, TB	Previous	2017	4	A few months	Mostly positive
P3	25	Man	White	Some graduate school	Urban	FB, TW, IG	Current	Mid-2018	1	Almost a year	Mostly positive
P4	20	Woman	South Asian	Some college	Urban	FB, TW, IG, SC	Current	2018	2	6 months	Mostly positive
P5	23	Man	White	Some graduate school	Urban	FB, TW, IG, SC, TB	Previous	Mid-2014	5 to 10	4 years	Mostly positive
P6	24	Woman	White	College	Rural	FB, TW, IG, SC	Current	Mid-2018	1	1 year	Mostly positive
P7	20	Woman	White	Some college	Urban	FB, TW, IG, SC	Previous	2018	1	A few months	Mostly positive
P8	18	Woman	White	High school	Urban	FB, TW, IG, TB	Current	April 2019	1	1 month	Mostly positive
P9	18	Nonbinary	White	High school	Rural	TW, SC, TB	Current	2014	4	2 years	Mostly positive
P10	20	Woman	White	Some college	Rural	FB, TW, IG, SC, TB	Previous	2017	2	4-5 months (still check in once in a while)	Mostly positive
P11	19	Woman	White	Some college	Rural	FB, IG, SC	Previous	Mid-2018	3	6 months	Mostly positive
P12	19	Gender-fluid	White	Some college	Rural	TW, IG, SC, TB	Current	2017	5	9 months	Mostly positive
P13	20	Woman	Asian	Some college	Rural	FB, SC, TB, RD^h^	Previous	2015	6	A couple of months	Both positive and negative

^a^Participants typed their gender and race, and we report the terms they used to describe themselves.

^b^BP: Buddy Project.

^c^TW: Twitter.

^d^SC: Snapchat.

^e^TB: Tumblr.

^f^FB: Facebook.

^g^IG: Instagram.

^h^RD: Reddit.

### Interviews and Data Collection

Participants included 10 women, 1 man, 1 nonbinary, and 1 gender-fluid person. The average age of participants was 20.3 (SD 2.25) years (range 18-25 years). In total, 6 participants lived in rural areas, and 7 lived in urban areas; 9 participants were White, 1 was Black, 1 was Latina, 1 was Asian, and 1 was South Asian. A total of 7 participants had previously used Buddy Project but were no longer active users and 6 were current users. The number of buddies ranged from 1 to 10, and the length of contact with a buddy ranged from 1 month to 4 years. A total of 11 participants described their experience with Buddy Project as being positive overall, whereas 2 described it as both positive and negative.

We conducted in-depth semistructured interviews that allowed participants to provide details about their experiences with their buddies while still covering topics regarding their experience with Buddy Project. The interviews were conducted via participants’ preferred method of voice or video call, and the audio was transcribed for analysis. The average length of the interviews was 47 min (range 28-56 min).

The interviews began with the interviewer sharing the goals of the study and asking for permission to record the conversation. The participants were then asked about internet and social media use before diving into their stories and experiences with Buddy Project. We asked to hear the story of how the participant found Buddy Project, why they started participating, why they continued or stopped connecting with their buddies, what the relationship with their buddy was like, and what topics they discussed. We asked questions about perceptions of the shared interest feature and their best and worst buddy experiences. We continued with questions regarding how their buddies compared with other friends, online and offline, and when relevant, they were probed about how they used Buddy Project to cope with mental illness or distress and how this compared with their other coping mechanisms (if any). It was important to us that participants only discussed experiences they felt absolutely comfortable with; therefore, we only probed on specific mental health experiences only if they mentioned the topic organically. We paid particular attention to a unique feature of Buddy Project, that is, the way buddies are matched based on the *interests* they select when they sign up. The interview protocol is available in Multimedia Appendix 2.

### Analysis

We started coding the data with an open coding procedure using Dedoose—a software for analyzing qualitative data. We followed the constant comparative approach [54], where we looked for patterns, consistencies, and differences in the data in an iterative manner. Specifically, one author coded one interview. Then, the 2 authors met with each other to discuss and refine the codes. The same author then coded 4 more interviews, for a total of 5, and the authors met to sort codes into themes and to further discuss and refine each code and theme. The same author then coded the next 8 interviews with these codes in mind, and the 2 authors met frequently to continue refining the themes. No further codes or themes emerged in the latter process.

## Results

We provide an overview of how participants connected with their buddies. Overall, participants used a variety of communication tools to connect with their buddies, including social media, text messages, video calls, and phone calls, with the most common being messaging and social media, which was most convenient for long-distance buddies so they did not have to pay data and messaging rates. These channels also protected buddies from releasing their phone numbers. One participant (P5) spent 2 weeks in person visiting her buddy. Communication frequency varied from multiple times a day to just enough interaction to stay in contact.

In the remainder of this section, we report 4 key themes that were derived from our analysis of participants’ experiences that contribute to Buddy Project’s success: building a support system for buddies, a stated and visible mission to prevent suicide in Buddy Project’s official online presence, matching buddies based on shared interests and identities, and not matching buddies based on shared diagnosis.

### Building a Peer Support System for Buddies

#### Buddies Providing Peer Support

Participants shared reflections on how buddies provided peer support. For example, P11 emphasized the importance of peer support and how Buddy Project contributes to making meaningful supportive connections:

I think [Buddy Project is] one of the most amazing things that there is, especially in terms of promoting your mental health...and having friends [that] actually are there for you and [Buddy Project is] trying to connect you to people with those interests [so that] you have those friends.

P11 gave an example of what the friendship with her buddy is like and how they exchange social support when she said the following:

They [the buddy] were definitely always trying to make sure the other person was happy no matter what the situation they just went through was… they were actually just very good about everything no matter how difficult a situation. They wouldn't go to bed unless you were okay and they were always there. That's just, I think, something everyone needs.

P8 touched on how her friendship with her buddy counteracts loneliness when she said: “I did feel more lonely before [having the buddy] ... But now I feel, I think, like I'm friends with someone.”

Referring to buddies as *friends* and these connections as *something everyone needs* are important demonstrations of what important gaps buddies fill in participants’ lives.

Buddies found that having the *option* to talk about mental health was important but not a necessary part of daily conversations with their buddies. Having someone who is simply there to talk about their struggles *as needed* is something that P5 found beneficial. She described the supportive relationship that she had with her buddy as follows:

Being there for each other when we need it, when we're both low. Knowing how to help each other feel better but just knowing that the other person is there if you just need to vent completely. They'll listen and understand and if they have anything to say, being able to say something that might help.

Similarly, P6 said:

I think it's important to have someone that doesn't have a bias, that shares in your issues. Her and I actually don't talk about mental health that much, but we both have this understanding that we both have issues, and we both want someone just to talk to when we need to.

Participants did not talk to their buddies about mental health challenges all the time nor did they want to; however, *knowing* that they *could* do so should they wish, was comforting and a unique aspect of buddies’ relationships with one another compared with their other relationships. These examples illustrate buddies providing emotional support (ie, communicating care and compassion) to each other.

Participants also noted sharing advice and tips, a type of informational support, to their buddies. For example, P9 described their relationship with their buddy as follows:

They got from the very basic, where do you live and whatnot, to asking about family life and being able to talk about like, “Oh, this is going on in my mental health region,” and giving each other advice on how to deal with it. And if things got too bad, they would stop me and be like, “Hey, you should talk to your parents about this or someone around you.”

Similarly, P13 noted the following:

I was giving the type of advice or comfort that I was trying to seek from other people. . .it’s how I help my friends. . .I did the same for my buddies.

#### Sense of Anonymity and Separation From Existing Known Ties

Participants noted that difficulties in disclosing mental health or other sensitive information with peers or friends (not buddies) included privacy concerns and not trusting that the other person would keep their information confidential. In contrast, with buddies, a sense of anonymity and separation of buddies from networks of known ties eased difficulties in feeling safe to share intimate information with others. P4 described it as follows:

If I were to come out to a friend, even if they're a friend I trust, there's still a part of this bigger circle that I'm in. They could always tell other friends, or they could accidentally slip up and tell a classmate, or a coworker, things like that. Whereas, my buddy, we're geographically is in a different realm. I know it'll stay between us and if it doesn't I don't have to worry about who they're sharing things with.

Peers may be unwilling to disclose their struggles with mental illness with known (not anonymous) peers in their social circles for a variety of reasons, such as the stigma surrounding mental illness (or talking about it), lack of helpful skills to effectively do so [55], or privacy concerns [56]. Buddies were often desired, in part, because they were perceived to likely be outside of one’s network of existing ties and without connections to one’s existing social network.

Although for some the separation from one’s network of known ties was helpful and provided a sense of desired anonymity, there were still other cases where the amount of afforded anonymity with buddies was insufficient. For example, P13 described how she used a completely anonymous forum as a mental health resource:

If I had a whole long thing that I needed to just rant about or get advice on but I wasn't comfortable talking to anyone in my life or even a buddy, I would go on the forum.

We see how although buddies were helpful, participants still felt the need to seek other resources to find the support they needed sometimes. Nevertheless, in this example, we see the unique position the buddy had in participants’ lives such that they could not talk about certain things *even* with a buddy. In this sense, a forum provided more anonymity than the buddy, whereas a buddy provided more anonymity compared with one’s in-person friends. As such, following previous work [57], we conceptualized anonymity as a continuum rather than a binary.

Although human connections can be supportive and helpful, they can also cause harm. Buddy Project provides participants with the Twitter or Instagram username of their peer match, which participants often used to ease worry about connecting with a total stranger. P10 noted the following:

It's an easy way to connect to people that's safer [than connecting with a random stranger] because you're connecting me to social media so you can see who the person is and you can know a little about them just by scrolling through profiles and it's not you're just talking to a random stranger.

Connecting with strangers on the internet is often a concern for those who seek online support [15]. When participants were able to gather cues on social media to assess the buddy, they felt safer to begin a conversation with them.

### A Stated Mission to Prevent Suicide on Official Buddy Project’s Online Presence

Mental illness can prevent people from feeling included and connected with peers, often categorizing those diagnosed as minorities, creating even more disconnection from one’s broader social environment. Overcoming stigma is often the first step to seeking and receiving support for mental illness [58]. Creating a safe space for discussing mental illness often requires a commitment to destigmatization, and Buddy Project is doing exactly that with their explicit mission to prevent suicide and commitment to raise awareness for mental health via their social media outlets. When asked what she thinks about the positive messages on Buddy Project’s Twitter and Instagram, P10 responded as follows:

I think it's great that they do that because not many people on social media are willing to just openly post about [mental health] so seeing the Wallpaper Wednesdays and all the positive messages... I think it's great that there is an organization out there that's willing to do that.

Relatedly, P3 described Wallpaper Wednesday as “weekly wallpapers for your phone and your mobile devices.” He continued as follows:

I think those are really awesome because you can set those up right away if you like the message, and you can set that as your screensaver or your background, and you have that positive message with you every day and at every single point that you need it.

He added the wallpapers remind him that his feelings were valid:

My feelings are valid. That's what I need this week.

Buddy Project’s mission and how it was exemplified in the organization’s social media accounts in practice had immediate importance to many participants.

The mission statement is the first piece of information that one sees upon entering the website, and the statement is embedded in both their Twitter and Instagram bios. P5 testified to the need for a safe conversation space when she said:

There's such a stigma around [mental health] to not talk about it with the people around you because you don't want to be judged. So, finding somebody on a platform where they advocate for mental health awareness just makes it different, and made me more comfortable to talk about it.

Echoing a similar point, P4 described how having a mission related to suicide prevention, or mental health, allows discussion of it to become less of a taboo topic with her buddies compared with other people she may meet online.

She said the following:

I feel like there are different boundaries in that ... The mission of Buddy Project is very directly related suicide prevention, that's not a taboo topic.

The explicit framing of Buddy Project allowed less perceived stigma between buddies to discuss mental health, resulting in more discussions of mental health compared with participants’ other social settings online and offline. We see how having a mission to raise mental health awareness and demonstrating that in action (eg, through providing resources or social media posts) is an important characteristic for technology-mediated peer-to-peer support systems that want to encourage participants to discuss mental health and exchange support.

### Matching Buddies Based on Shared Interests and Identities

We found that another feature that participants deemed helpful with Buddy Project was how peers are matched on the basis of shared interests and identities (eg, music, television shows, identity). Our analysis suggests that shared interests act to promote conversation between new buddies who are otherwise technically 2 strangers at the time of being paired. In addition, salient shared identities help with ensuring that one will be understood when discussing mental health and intersections of their identities with mental health.

#### Shared Interests as Conversation and Compatibility Aid

Matching buddies on the basis of shared interests had several perceived benefits and functions for participants, which stem from the central theme that shared interests act as support for better conversations, leading to deeper connections at a higher pace. This way of being matched acted as a means for starting conversations, as P3 described:

I think [the shared interest is] good because it gives you something to talk about. If you didn't have a shared interest I think it would be a little harder to start up conversations whereas if you have that common interest you can be like, ‘What did you think about this part of our common interest?’ And then you can start a conversation from there and then it can drift off into other things about your life.

Similarly, P10 described her experience with using the shared interest to start conversation:

It made it easier to talk to them because you went in with this baseline interest that you can both just start talking about and it wasn't awkward icebreaker conversations... you get that instant connection and you understand already why you are talking to this person as opposed to if you're meeting somebody in person for the first time you don't always know what to talk about

She continued to describe the process:

If I message them first, I'll just go in with, “Hey, our interest is this. What do you like about it or what do you know about it?” And just to start the conversation where it's not awkward back and forth like, “Hi, how are you?” Because those conversations can get tedious and boring.

In this way, shared interests mitigated the risk of potentially losing out on relationships that have the potential to be helpful because buddies can begin their relationship with discussing topics that are meaningful to them (and are not necessarily related to mental health) rather than topics that they do not share an interest in.

Participants also noted that the shared interest feature improves the likelihood that one will find a compatible companion. P1 described this as follows:

If I saw something that I thought was cool I could send it to her to also see, or if she saw something she could send it to me and then that would just keep our conversation going.

The shared interests made it more likely that participants were matched with a compatible buddy. They acted as optional conversation prompts and facilitated the formation of deeper connections between buddies.

#### Shared Identity to Ensure Being Understood When Discussing Mental Health

Participants noted that it was more helpful to connect with someone who shared a salient identity with them—identities that they perceived to shape their mental health experiences. For example, P6, a self-identified neurodiverse person, said as follows:

I think it's definitely easier [to connect to another neuro-diverse individual to discuss mental health]... I have one friend in real life [also neuro-diverse] that her and I say people who are neuro-typical … just don't understand necessarily sometimes the issues that we have or that we're going through.

She continued:

I think it's easier to talk to someone who kind of gets it a little bit, given I would talk to anyone that had any issues... I think it's really helpful to talk to someone, and have someone else just get it.

P6 found it easier to talk to neurodiverse peers, especially about mental health, compared with others who did not share this identity facet with her. In fact, many neurodiverse individuals consider neurodiversity as part of their identity, not an illness [59].

As another example, P12 described how Buddy Project “helps you find people ...Especially with pan[sexual], it helped me find people that are like myself.” As a result of having a buddy with similar identities, participants felt that they would be understood and seen within their buddy relationship.

### Not Matching Buddies Based on a Shared Mental Health Diagnosis

Participants’ accounts suggest that shared mental health diagnosis may not always be the best way to match peers who need social support to cope with mental illness. We found that buddies typically base their first conversations off their stated shared interests. As previously described, buddies’ experiences have been positive with the shared interest feature, whereas the mental health orientation of Buddy Project creates a shared understanding that discussing mental health with buddies is not taboo. Participants noted that when the shared diagnosis replaces the shared interest, the dynamic of the relationship changes. The friendship aspect is lost, as discussing mental health diagnosis is not how people begin to form friendships with one another; of course, once a relationship exists, discussing mental health is something participants feel more comfortable with. In this section, we describe how participants reflected on the perceived benefits and drawbacks of being matched with someone that shares their same mental illness diagnosis. These include concerns about differences in the severity of buddies’ experiences, engaging in self-comparison, and sharing unhealthy coping mechanisms.

#### Comparing Self and Severity

According to participants, one of the perceived drawbacks of being matched with someone based on a diagnosis is that mental illnesses can vary in severity. P11 described this drawback as follows:

The cons would be just the different levels of each different type of mental illness. Anxiety has so many different levels that sometimes you really can't connect because someone's [level of severity] could be so low and then someone else's [level of severity] could be so high.

As a result of different severities of anxiety, participants’ symptoms can be so varied that it could hardly look like the same condition. It is likely that if 2 people are paired with the same mental illness, they are expected to be able to connect over the diagnosis and experiences. When this does not happen because of different severity levels, participants may become discouraged from using peer-to-peer support or not find the validation and support that they need, making mental health support resources scarce.

Participants also noted that matching based on diagnosis might lead to self-comparison on coping and wellness between buddies. For example, P12 described the competitiveness that self-comparison may cause:

I guess if you're talking to someone who's had a similar diagnosis as me but their experience was still different, I feel like sometimes it would become a game of “Oh, mine is worse” or that kind of game.

When individuals compare the severity of their mental illness with that of someone who seems to be coping more effectively than they are, one is subject to negative self-talk [60], which is not helpful in coping.

On the other hand, when participants share the same diagnosis and the same severity level, they will often share similar experiences that allow for a deeper understanding of each other. P6 spoke to the point that anxiety varies in severities and that a benefit of connecting based on shared diagnosis and severity is that one is more likely to find someone who truly understands their experience. P6 said that she would rather connect with someone that has the same diagnosis *and* severity level than someone who just has the same diagnosis broadly:

I think it's better to be able to just connect with someone on that [severity] level, just because they do truly understand what you're going through or how you're feeling...

Variations in severity can lead one to compare their mental state with their peers, which is not always a helpful coping strategy. However, sharing the same severity of mental health diagnosis can result in a deeper understanding for one another and less competitiveness. Overall, for participants wanting to connect with others over a similar diagnosis, severity level was a criterion to ensure the connection would be helpful. Further research is required to examine how the design of peer support technologies would account for fluctuating severities. In this study, we provide preliminary evidence that is a relevant criterion to consider.

#### Sharing Unhealthy Coping Mechanisms

Another potential downfall to matching buddies based on shared diagnosis is the risk of sharing unhealthy coping mechanisms. P10 gave an example of sharing unhealthy coping mechanisms when asked about being matched with a buddy with the same condition:

If you sleep all day to help you cope with something, telling the other person that they might try sleeping more to see if that helps them. Not the healthiest option, but it's what you do.

Other unhealthy coping mechanisms may include self-harm, substance abuse, changes in diet, and a disconnection from reality. These negative changes can result in decreased mental health [61], which defeats the purpose of any peer support system.

Overall, participants’ accounts highlight the nuances that shared diagnosis matching may have, sometimes leading to more understanding and sometimes leading to unhealthy coping mechanisms or otherwise unhelpful behaviors such as self-comparison.

## Discussion

### Principal Findings

Although Buddy Project is not the most technically sophisticated peer support system, we found that its design principles are effective in facilitating relationships that help young adults cope with mental illness by engaging in peer support. By taking Buddy Project as a case study that uses technology to pair up young people to cope with mental illness, we provide insights into the qualities possessed by effective technology-mediated peer support systems. We found that designing a technology-mediated peer-to-peer support system with a commitment to mental health awareness is an effective way to create a coping tool for young adults facing mental health challenges. This clear commitment lowers the stigma attached to mental health discussions between buddies and provides a shared understanding that sharing mental health–related experiences is a legitimate need that can be met within this peer support system.

Future digital peer support tools for mental health can use a combination of the features that Buddy Project users found helpful: (1) semiguided chat spaces made possible through matching based on interests and identities that peers self-identify with and having an explicit mental health–related mission coupled with social media and other online presence that makes it clear that discussing mental health is safe within the peer support ecosystem; (2) not matching based on broad mental health diagnosis; however, if diagnosis is important to peers to incorporate such matching with educating peers on how to provide helpful support and how to avoid unhelpful coping mechanisms and accounting for symptom severity in matching; (3) allowing for some degree of anonymity and control over how peers present themselves to each other; (4) allowing potential peers to assess the fit and whether they would like to embark on a relationship with their peers based on browsing public social media profiles of one another or innovating privacy-preserving means of assessing initial fit and trust. We describe these in detail in the remainder of this section.

### Semiguided Chat Spaces for Mental Health Coping and Developing Meaningful and Supportive Relationships

#### Mental Health–Related Mission

We found that having an explicit message such as Buddy Project’s mission to prevent suicide creates a space where peers feel safe to talk about mental health; however, mental health is not all that they feel compelled to talk about. Removing people’s fear of being judged by their illness not only contributes to more fluid communication but is an essential element of a trustworthy relationship [62]. Once people recognize their condition and are able to communicate their experiences with others, they are taking the first step to recovery and are more likely to seek further help [16]. We recommend that future peer support systems and organizations aiming at facilitating mental health peer support make it an explicit point to advocate for mental health destigmatization through all their outlets in practice.

#### Shared Interests and Identities

Although the mental health mission provides a stigma-free space to discuss mental health among peers, buddies found that being matched based on shared interests and identities (and not diagnosis) provided complementary context for them to develop deep meaningful friendships in which discussing mental health *and* other topics were welcome.

#### Semiguided Conversation Space

Taken together, shared interests and identities and the mental health mission statement create what we call a *semiguided* conversation space: an open and flexible yet somewhat guided conversation space, ideal for connections that are both deep and supportive. We describe how we came to this notion in the remainder of this section.

O’Leary et al [24] designed a chat system in Google Docs to study the impact of guided and unguided chats for technology-mediated mental health peer support. In their study, the guided chats followed a script, whereas the unguided chats had no prompts at all. They found that guided chats resulted in deep connections, where peers provided solutions to problems and new perspectives. Unguided chats resulted in smooth conversations that offered personal connections. Their participants referred to unguided chats as *pleasant and relaxing* acting as a distraction, or temporary relief, from one’s problems. We found that chats with buddies fell somewhere in between what O’Leary et al [24] referred to as guided and unguided chats, which we call a *semiguided* conversation space.

We describe semiguided chats as those that have the characteristics of both guided and unguided chats to some extent. Semiguided chats are similar to guided chats because of the way the shared interests or identity and mission statement guide conversations among buddies; they are different in that they do not use explicit prompts for buddies to engage in conversations about as guided chats do. Unlike a fully unguided chat space where this shared understanding is missing, the sociotechnical space with semiguided chats is not completely void of possible topics that peers would know they can safely speak to their buddies about (eg, shared interests or identities, mental health). We found that the semiguided conversation space provided by Buddy Project facilitates the development of meaningful friendships and personal connections and a supportive context within which discussing mental health was safe and comfortable and where buddies would exchange stories and perspectives to help each other. However, buddies did not feel that they needed to discuss mental health all the time nor did they feel that they absolutely needed to provide actionable advice to their peers in contrast to what a guided chat space would require. We suggest that a semiguided chat space will allow peers to not only gain the benefits of both guided and unguided chats but will also likely not evoke unwanted feelings for buddies (eg, an unwanted sense of responsibility to help their peers, as was the case in the study by O’Leary et al [24]).

Semiguided chats offer buddies the option to fluidly switch between deep and light conversations, whereas unguided and guided chats [24] were focused on one or the other. Semiguided chats allow peers to find the middle ground between exchanging social support and problem solving, which could allow peers to use both mechanisms in one resource (ie, relationship with the buddy). We suggest that semiguided chats are a good design principle when creating a sociotechnical space for mental health discussions and peer support. They allow peers to have freedom in what they talk about so the conversation does not feel forced, yet they are free flowing. Combined with a message about mental health, the conversation will still likely gravitate toward becoming an effective coping tool, as it did for Buddy Project users participating in our study.

Previous research has recommended connecting peers in online communities on the basis of similar features other than diagnosis in contexts such as breast cancer [63], various cancer diagnoses [64], and caregivers of individuals with cognitive illnesses [65]. Specifically related to mental illness, recent work [24] suggests that technology can enhance peer support for mental health by matching peers based on similarities that go beyond diagnosis. However, this previous research begs the questions of what this peer support might look like in practice, what peers’ attitudes toward it might be in practice, or what other features are important to make peer support matching helpful to peers. Buddy Project’s design, as we found, fills this gap in our knowledge.

### Striking a Balance: Matching Peers Based on the Mental Health Diagnosis or Not

The participants in this study expressed concern about the potential costs of being matched based on shared diagnosis—comparing one’s self and severity of illness and sharing unhealthy coping mechanisms. In fact, individuals with mental illnesses do not always prefer for their peers to have the same diagnosis as theirs [15]. This finding resonates with previous studies, suggesting that sharing unhealthy coping mechanisms is one of the most likely disadvantages of online support groups and peer support [38,55,60,66]. This can include suicidal ideations being shared among peers, and even the possibility of a suicidal pact, and an overall diminished self-esteem and well-being [55]. In the case of depression, which affects an estimated 25% of young adults [67], hearing a peer’s depressive thoughts can cause a downward spiral [56]. Being matched based on the same diagnosis can also lead to lower self-esteem and hope when people engage in self-comparison and feel as though others are doing better [16], which counteracts the peer support that one should be receiving [17].

However, with the right system and the right peer match (eg, facilitated through shared interests and identities), sharing healthy coping mechanisms could be a positive experience for participants as it acts as an outlet to seek and provide helpful mental health resources and provide a sense of solidarity and connection to peers [22]. For example, if a participant has been seeking help from a therapist, they could suggest their peer to do the same and support their claim with personal experience, making it more likely that their peer seeks additional mental health support. For this to be possible, we argue that individuals would have to be educated on what healthy and unhealthy coping mechanisms are. In addition, when matched based on diagnosis, peers can provide support to each other around common stressors, symptoms, stigma, and other challenges, resulting in them feeling less alone [58].

A useful approach that addresses the concerns and allows users to receive the benefits of shared diagnosis pairing could be offering educational material to peers that explains to them the potential dangers of these unhealthy actions and instead educating them on healthy coping mechanisms. There is further evidence for an unmet demand for such educational programs, as many individuals express interest in training to become a peer counselor [68]. For instance, the 7 Cups of Tea website educates users on active listening and support provision; similarly, Crisis Hotlines trains their volunteers.

Technological spaces such as Buddy Project or other services dedicated to mental health advocacy and awareness can use their online presence (eg, social media posts) to provide such educational material. They can also provide a set of resources to buddies once they are matched, educating them on healthy coping mechanisms and how to be a supportive, compassionate buddy. The effectiveness of any such approach should be investigated in future research. Our study provides initial insights that there is room for improvement when it comes to engaging in healthy behavior in technology-mediated peer support systems for mental health when a shared diagnosis is a factor in matching peers.

### Anonymity, Privacy, Intimacy, and Safety in Relationships Between Buddies

Privacy concerns and the risk associated with sharing information are key challenges for internet users because they can make them more reluctant to sharing information, asking for support, and building connections with other users [69]. It can also be difficult to reach out to strangers with whom one has no pre-existing ties to find support. One of the reasons Buddy Project is a popular choice among young people seeking online peer support is the sense of anonymity and separation from participants’ existing and known networks (eg, family, classmates). They are not completely anonymous, as buddies have access to their peers’ Twitter or Instagram account and share varying levels of personal information with each other; however, there is a sense that they are unlikely to have a pre-existing tie with their buddy. Therefore, their buddy would also not know anyone in their existing social network. There are also no requirements for sharing physical world names or identities, as they are in platforms such as Facebook. As a result, buddies feel they can safely share their mental health struggles in confidence, without worrying about others’ undesired access to their personal information and struggles.

A sense of anonymity facilitates openness in self-expression, support seeking, and support provision around sensitive topics [70-72]. Our findings suggest that control over how to present oneself (eg, using a pseudonym or just the first name) coupled with separation from networks of known ties (ie, existing social connections such as friends and family) and some contextual information through access to the prospective buddy’s social media accounts, or shared interests, helped peers to take the first steps in a relationship with a buddy. Once there was more trust developed in the relationship, participants revealed more and deeper information about themselves, consistent with the social penetration theory [73]. This theory suggests that relationships develop and become more intimate over time as people share more intimate information with each other and move away from solely shallow information [73]. Here, we see how access to prospective buddies’ social media profiles before conversing with them for the first time in tandem with the mental health mission and shared interests matching provide a fruitful sociotechnical space for developing deep supportive relationships with buddies.

Giving individuals who sign up to be paired an option to add their social media information is how Buddy Project conducts its matching process; however, users do not *need* to have *public* profiles. We learned that being able to assess their proposed buddy before connecting with them through the availability of some signals on their social media was helpful to participants. However, this would be harder for those whose online profiles are private or do not include much information. We suggest that Buddy Project or similar systems provide ways for their users to assess their buddies before deciding to connect; this does not have to occur using social media platforms. For example, an alternative would be creating a profile within the system that has an *about me* page, how long the person has been a user, buddies paired with, and any other information that users choose to share about themselves.

Some drawbacks of connecting online to find peer-to-peer social support are privacy concerns and cyberbullying [31]. Our findings demonstrate privacy concerns but not much about cyberbullying concerns—an important area for future research. In summary, our findings suggest that peer support systems should consider allowing some degree of anonymity, separation from existing social networks, and control over presentation, along with providing tools to assess and maintain safety and initial fit with prospective buddies before connecting with them.

### Conclusions

We contribute to an understanding of desirable features for digital mental health peer support systems: (1) matching peers on the basis of interests and identities they identify with; (2) having an explicit mental health mission coupled with other online presence to signal that discussing mental health is not a taboo within the digital peer support system; and (3) not matching peers based on broad mental health diagnosis; however, if diagnosis is crucial to account for, accounting for illness severity and educating peers on how to provide support while avoiding suggesting unhelpful coping mechanisms; (4) allowing for some anonymity and control over how peers present themselves to each other; and (5) providing relevant information and tools to potential peers to aid in their decision in connecting with the proposed peers before connecting with them.

### Limitations and Future Work

As is common in interview studies, this study’s sample was not representative of Buddy Project users or their experiences with the service, even though we sought a diverse participant group in demographics and experiences. Future work is needed to evaluate our findings with a larger and representative population, possibly through other methods (eg, survey of Buddy Project users). Nonetheless, following best practices in interview research, our goal is not generalizability [74], rather generating conceptual insights. The challenges faced by individuals with multiple intersecting marginalized identities are worthy of future exploration. For example, although we note that connecting over identities is helpful, future research should explore what an ideal connection would look like when multiple identity facets are concerned.

Our study does not account for all kinds of mental illnesses, and further research is needed to examine the similarities and differences in designing for various mental health peer support systems. Nevertheless, we identify the factors that such designs should consider.

Within our interview participants, we did not encounter anyone who mentioned that they had encountered fake profiles or those with malicious intentions. However, this does not mean that such harmful interactions do not happen. It is possible that those willing to interview with us had more positive experiences. For example, Buddy Project shared our study link on their social media accounts. It is possible that those who follow Buddy Project on social media have had better experiences with the platform (although we attempted to recruit individuals with both positive and negative experiences). However, examining how to design technologies to facilitate trust and safety while reducing harmful behavior is an ongoing area of research that is beyond the scope of this study.

## Appendix

Multimedia Appendix 1 Buddy Project's interests and identity options.

Multimedia Appendix 2 Interview guide.
