# Supplementary material for: Considerations in Designing Digital Peer Support for Mental Health: Interview Study Among Users of a Digital Support System (Buddy Project)
Source: JMIR Ment Health. 2021 Jan 4;8(1):e21819. doi: 10.2196/21819 (PMC7813628; doi:10.2196/21819)
Supplement: Multimedia Appendix 1 [file mental_v8i1e21819_app1.docx]

### Interests and Identities

We used the Buddy Project’s sign up process to gather a list of all the available “interests” that users have the option to pick from when signing up to be paired with a buddy. We then iteratively produced higher level categories (e.g., performing arts, musicians, music genres, gender and sexuality) that these interests belong to. Then we created the larger broad categories of interests and identities (i.e., arts and entertainment, identity, time zones).

Arts and Entertainment

Performing Arts: Dear Evan Hansen, Hamilton, musicals, singing/performing, theatre/musicals

Musicians: 5 Seconds of Summer, Alessia Cara, All Time Low, Ariana Granda, A$AP Rocky, Bazzi, Billie Eillish, BROCKHAMPTON, Bronnie, BTS, Demi Lovato, Dodie Clark, EDEN, Fall Out Boy, Halsey, Harry Styles, Hayley Kiyoko, Hippo Campus, Jacob Whitesides, Kali Uchis, Lady Gaga, Lana Del Rey, Led Zeppelin, Linkin Park, Logic, My Chemical Romance, One Direction, Panic! At The Disco, Pink Floyd, Post Malone, Rex Orange County, Shawn Mendes, SZA, Taylor Swift, The 1975, The Beatles, The Neighbourhood, Twenty One Pilots, Tyler The Creator, Waterparks, Why Don’t We

Music Genres: alternative music, country music, EDM, Indie Music, Kpop, pop music, pop punk music, rap music

Visual Arts: filmmaking, horror films, makeup, painting/drawing, photography

Television: American Horror Story, Big Brother, Criminal Minds, Game of Thrones, Grey’s Anatomy, New Girl, Queer Eye, Riverdale, Stranger Things, Super Natural, The Office

Youtubers: Dan & Phil, Dolan Twins, Jacksepticeye, Kendall Rae

Actors: Froy Gutierrez

Companies: Disney, Marvel Comics

Literary Arts: anime, comic books, poetry, reading, writing, young adult literature

Books: Harry Potter

Sport/Games: dancing, fitness, football (American), football/soccer, gaming, hockey, horseback riding, pro wrestling, sports

Other: animals, art, baking/cooking, coding, interest doesn’t matter, memes, podcasts, STEM, traveling, Youtubers

Identity

Religion: Buddhism, Christianity, Hinduism, Islam, Judaism

Gender and Sexuality: asexual, bisexual, drag race, gay, lesbian, LGBTQ+, non-binary, pansexual, transgender

Race and Ethnicity: African, African-American, Native American, Afro-Caribbean, Afro-Latinx, Arabic

Country: Australia, Canada, Central Asia, East Asia, Africa, Europe, Mexico, South America, South Asia/Desi, Southeast Asian, United Kingdom

Political or other Ideology: activism, conspiracy theories, feminism, vegan/vegetarian

Communities: Spoonie/PWD

School Year: college freshman/first year, college junior/third year, college senior/fourth year, college sophomore/second year, high school freshman/9th grade, high school junior/11th grade, high school senior/12th grade, high school sophomore/10th grade

Time Zones

Central Standard Time, Eastern Standard Time, Mountain Standard Time, Pacific Standard Time
