# Supplementary material for: Considerations in Designing Digital Peer Support for Mental Health: Interview Study Among Users of a Digital Support System (Buddy Project)
Source: JMIR Ment Health. 2021 Jan 4;8(1):e21819. doi: 10.2196/21819 (PMC7813628; doi:10.2196/21819)
Supplement: Multimedia Appendix 2 [file mental_v8i1e21819_app2.docx]

### Interview Guide

Introducing the study and summarizing its goals, asking what the participant would like to be referred to as, and if we have permission to record the conversation.

The following topics guided interactions with interview participants:

What are the top 3 things you do on your phone online? How about on your laptop? Why?

What about social media? Where do you spend most of your time? Why?

What kinds of things do you post on each of these platforms? What kinds of things you don’t post? Why?

Do *you* talk about mental health on these platforms?

Do you have a preference for a certain app? Why?
Who are the people you connect with on each platform? Why?

Tell the story of:

How you found the Buddy Project.

Why you started participating.

Why you kept coming back.

What it was like.

How many buddies you’ve had.

What interests did you select at the time to find these buddies? Why?

Did your shared interests help you connect with your buddy?

Best and worst buddies.

- Why was this a good experience? Why was this a bad experience?
- Duration of contact (one day/one week/one year/etc.)
- Type of contact (in person/online only/etc.)
- What kinds of things did you talk about?
  - What is the significance of your selected interests? (this question was added after the second interview)
  - Did you share any of your worries/concerns/problems with your buddy? How did they respond? Were they able to help you resolve it/come up with a solution?
  - Was your buddy able to give you a new and encouraging perspective on the issue?
  - Did your buddy share any of their worries with you? How did you respond? Were you able to help resolve the problem/come up with a solution? Give them a new perspective?

Shared diagnosis (if relevant)

- Do you and your buddy share the same diagnosis?
- What do you think the pros and cons are of talking to someone with the same diagnosis versus a different diagnosis?
- Do you have a preference on whether or not you share the same diagnosis with your buddy?
- What kinds of things did you not talk about?
  - Did your buddy ever tell you something that disturbed you? Did you ever feel like you were responsible to help them with something that made you uncomfortable?
- What makes for a good buddy for you? Why?
- What makes for a bad buddy for you? Why?
- Why are these traits important to you?

How do you feel like your relationship with your buddy has changed over time?

- For example, with respect to the kind of things you talked or did not talk about
- Time of the day?
- Other changes?

Have you ever looked for or found someone else to talk to online? Why did you do so?

- Tell me about a good experience. Why was it good?
- Tell me about a bad experience. Why was it bad?

e.g. dating apps, support groups, forums, anything on the internet really

How were those experiences the same or different from the Buddy Project?

How about other social media sites you use?

Mention something difficult (this section was added after the first interview):

- Was the BP helpful? How was it helpful?
- How did it differ from in-person connections or other social media sites?

Mention talking to their buddy about mental health struggles (this section was added after the first interview):

- How do you think having this talk through BP was different from in-person connections or other social media sites?
- How did BP fit into how you cope with mental health struggles?

How has the Buddy Project been different from or similar to your in-person daily connections?

Matching (this section was added after the first interview):

- What do you think about this way of being matched based on interests? What do you think are the pros and cons? Why?
- Are there other ways you imagine being matched on would be helpful to you?

Would you recommend The Buddy Project to others? Why/why not?

How would you describe the Buddy Project to someone?

Has your perspective of the Buddy Project changed over time/since you started using it? How?

To conclude, we provided information about the gift card and how they can get in touch with us after the interview.
